# Hybrid Management in patients with complex aortic pathology - single center experience

**DOI:** 10.1186/1749-8090-10-S1-A49

**Published:** 2015-12-16

**Authors:** Dimitar Petkov, Dimitar Kyuchukov, Rumen Iliev, Gencho Nachev

**Affiliations:** 1 Department of Cardiac Surgery, "St. Ekaterina" University Hospital, Sofia, Bulgaria 1431

## Background/Introduction

Some cardio-vascular diseases are still a therapeutic challenge. They cannot be treated only by surgeons or only by interventional cardiologists. These difficult cases need a combined team and combined procedure - so called "Hybrid procedure".

## Aims/Objectives

The objective of this presentation is to provide an overview of the experience of our center with hybrid management of high risk patients with complex aortic pathology.

## Method

From 2003 to 2015 a total number of 84 patients, 63 male and 21 female with average age 51.7 years (from 26 to 86 years), underwent hybrid management of complex aortic pathology. Patients were divided in 5 groups: Group 1: Hybrid aortic debranching in 12 pts (for Aortic dissection type B with retrograde arch dissection in 10 patients and Aortic re-dissection type A after surgical treatment - 2 patients); Group 2: TEVAR after conventional surgery- 47 patients (for treating Aneurysm after patch correction for coarctation - 13 patients and Re-dissection after ascending aorta or arch surgery - 34 patients); Group 3: Conventional surgery after TEVAR- 13 patients (Second-stage procedures: Bentall/DeBono - 4 patients; Wheat - 2 patients; Arch replacement - 3 patients; Surgery for complications - 4 patients: Aneurysm sac rupture - 2 patients. Severe endoleak "type 1" - 1 patient. Acute severe limb ischemia due to left a. subclavia closure - 1 patient); Group 4: Bentall/De Bono procedure plus TEVAR (single stage)- 1 patient; Group 5: Second-stage TAVI after coronary surgery-11 patients.

## Results

One patient died in Group 1, because of aortic rupture. Two patients died in Group 2 TEVAR after conventional surgery, because of aortic dissection complications - one from intestinal ischemia and one from bronchomalacia. Multiple organ failure is cause of death in three patients from group 3 and one patients in group 2. One patient in Hybrid arch debranching group received stroke. All other patients had uneventful recovery and were discharged home.

## Discussion/Conclusion

Our experience shows that hybrid management in patients with complex aortic pathology is a reasonable approach for management with acceptable morbidity and mortality. Hybrid management could be recommended as optimal treating strategy for high risk patients.

